# Moxibustion inhibits inflammation in monosodium urate crystal-induced gouty arthritis model rats through metabolomic regulation

**DOI:** 10.3389/fmolb.2025.1433912

**Published:** 2025-03-03

**Authors:** Yufeng Xie, Yun Chen, Ting Qin, Jun Li, Zhichun Chang, Yanfang Li, Jianmei Zhang, Mujun Liu, Jianli Wang, Rong Ren, Ziliang Qian, Jinxin Liu, Min Chen

**Affiliations:** ^1^ Faculty of Chinese Medicine and State Key Laboratory of Quality Research in Chinese Medicines, Macau University of Science and Technology, Macau, China; ^2^ Shenzhen Hospital (Futian) of Guangzhou University of Chinese Medicine, Shenzhen, China; ^3^ The Sixth Clinical Medical College, Guangzhou University of Chinese Medicine, Shenzhen, China; ^4^ Shenzhen Graduate School of Peking University, Guangdong, Shenzhen, China

**Keywords:** moxibustion, gouty arthritis, inflammation, metabonomics, LC-MS

## Abstract

**Background:**

Moxibustion is a form of therapy that to warm the acupoints located skin by using dried mugwort leaves. It is widely used to treat gouty arthritis (GA). However, the mechanism of moxibustion on improving GA has not been fully revealed. In this study, we explore the mechanism of moxibustion on GA via metabolomics combined with traditional Chinese medicine (TCM) theory.

**Methods:**

Three days before model induction, the rats of moxibustion groups were treated with moxibustion in the ST36 and SP6, and then, a GA rat model induced by monosodium urate (MSU) was established. Biological samples, including joint synovial tissue and serum samples, were collected and measured by histopathological staining, molecular biology assays and liquid chromatography-mass spectrometry (LC-MS)-based metabolomics.

**Results:**

We found that moxibustion could reduce the ankle edema induced by MSU crystals, decrease the expression of related proinflammatory genes, decrease the levels of serum IL-18 and IL-1β, and restore the metabolism of glycerol phospholipids, niacin and nicotinamide in GA model rats.

**Conclusion:**

Moxibustion can regulate the metabolism of GA model rats widely to inhibit inflammation. Our research deepens our understanding of the complex mechanisms of moxibustion and promotes the application of moxibustion in the clinical practice.

## 1 Introduction

Gouty arthritis (GA) is a common inflammatory arthritis characterized by pain, swelling, limited movement. Inflammation of the affected joints can lead to hypertension, diabetes, arteriosclerosis, and uremia (Hao et al., 2020). According to an epidemiological survey, the worldwide prevalence of GA is approximately 2.7%–6.7% (Chen-Xu et al., 2019; Zobbe et al., 2019; Rai et al., 2017; Kuo et al., 2015; Zhu et al., 2011; Anagnostopoulos et al., 2010). Moreover, according to retrospective data from the Global Burden of Disease, the prevalence and incidence of gout has increased in 204 countries and regions from 1990 to 2019 (Jeong et al., 2023). The pathogenesis of GA mainly involves the elevation of blood uric acid concentrations and the deposition of uric acid crystals in the joints, which can cause inflammatory pain. At present, therapies that control uric acid levels and inflammation are available for the treatment of GA. Although the anti-inflammatory drug including colchicine, nonsteroidal anti-inflammatory drugs, and glucocorticoids have been used to treat patients with GA, these medications have side effects such as gastrointestinal reactions, nephrotoxicity, liver damage, and other adverse events (So, 2008). Long-term pain and drug side effects severely affect the daily life of patients with GA. Consequently, the development of effective replacement therapies for GA is urgently needed.

Moxibustion is a mainstay treatment for diseases in ancient China. It is a heat therapy that directly acts on acupoints and has been shown to regulate inflammation in many studies. In China, moxibustion is widely used to treat inflammatory arthritis, including GA (Gao et al., 2015; Xiao et al., 2017; Zhang et al., 2013; Gao et al., 2017). There are two main mechanisms by which moxibustion regulates inflammatory reactions, including the direct and indirect mechanisms. In terms of the direct mechanism, moxibustion therapy can act on the hypothalamus-pituitary adrenal axis, affecting the expression of glucocorticoid receptors and regulating the secretion of melatonin. In terms of the indirect mechanism, moxibustion therapy can regulate inflammatory factors and reduce inflammatory reactions via the JAK-STAT and NF-κB signaling pathways (Galligan et al., 2010; Zhang et al., 2017). David Julius, the winner of the Nobel Prize in Medicine or Physiology in 2021, discovered the capsaicin-specific receptor transient receptor potential vanilloid 1 (TRPV1) (Caterina et al., 1997). TRPV1 is activated at temperatures exceeding 43°C, and moxibustion may activate the TRPV1 pathway to induce local endothelial cells, mast cells, keratinocytes, and other cells to produce a variety of cytokines and other active substances, resulting in local anti-inflammatory effects (Huang et al., 2017). However, the 2020 American College of Rheumatology guidelines on the prevention and treatment of gout recommend the use of local ice compresses as an alternative treatment (FitzGerald et al., 2020). The mechanism of moxibustion inhibits inflammation by regulating metabolic pathways has not been reported.

Metabolomics has unique advantages for understanding the overall therapeutic effects of moxibustion and further elucidating the differences between different methods by identifying the relevant metabolic changes (Hagenbeek et al., 2020). GA typically occurs in the joints of the body, and the exchange of molecules through the circulatory system also plays a role in the pathogenesis of GA. Therefore, joint histopathology and molecular biology methods can be used to reveal specific localized responses to stimuli in the joint, and serum metabolomics analysis can provide complementary information from surrounding tissues. In this study, liquid chromatography–mass spectrometry (LC-MS)-based metabolomics of serum and other molecular biology techniques was used to observe the therapeutic effects of moxibustion and colchicine on GA model rats and the underlying mechanisms. Overall, our study deepens our understanding of the complex mechanisms of moxibustion and promote the application of different methods in clinical practice.

## 2 Materials and methods

### 2.1 Experimental animals

Male SD rats weighing approximately 180 ± 20 g were purchased from GemPharmatech Company Limited (Guangdong, China). The animal study protocol was approved by the Animal Care and Use Committee of Peking University Shenzhen Graduate School (Permit Number: AP0044001). All procedures were conducted in accordance with the regulations of the “International Council for Laboratory Animal Science”.

The rats were randomly divided into 4 groups (6 rats per group): the control (Con) group (the group that did not receive any specific intervention in the experimen), acute gouty arthritis model (AGA) group, moxibustion-treated (Mox) group and colchicine-treated (Col) Group. All animals were maintained under controlled environmental conditions (21°C ± 1°C, relative humidity of 65% ± 5%, and 12 h light/dark cycle) with free access to water and food. After 3 days of adaptation, all rats except for the control rats were subjected to AGA modeling via injection to the right ankle with 0.3 mL 5% monosodium urate (MSU) (Coderre and Wall, 1988). The control rats were administered the same amount of saline during the experimental period.

### 2.2 Moxibustion and colchicine treatment

**FIGURE 1:**
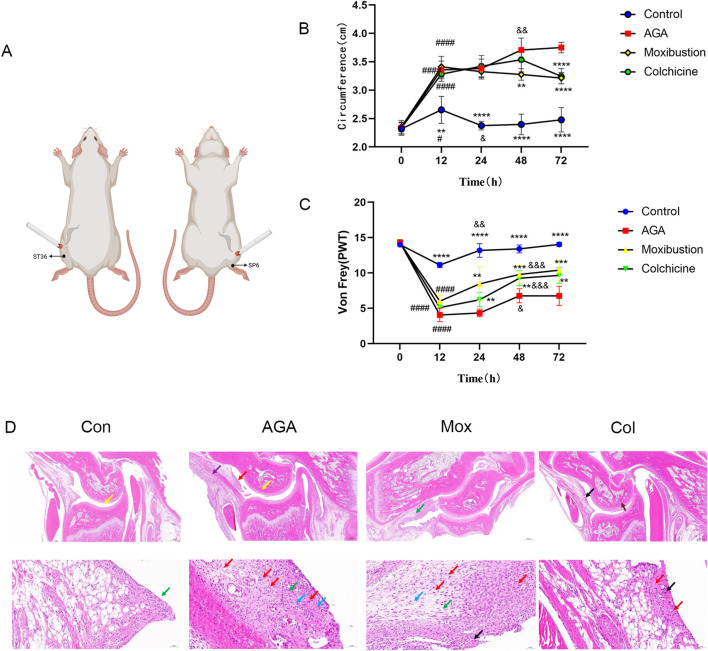
Moxibustion reduced joint inflammation in MSU-induced GA model rats. **(A)** Experimental conditions for the moxibustion treatment. **(B, C)** Right ankle edema and the PWT of rats in the 4 groups at different time points. (Time 0: immediately before molding, Time 12/24/48/72: 12/24/48/72 h after modeling, ****P < 0.0001, ***P < 0.001, **P < 0.01, *P < 0.05 indicate significant differences vs. the AGA group. ^####^ *P* < 0.0001, ^###^ *P* < 0.001, ^###^ *P* < 0.01, ^###^ *P* < 0.05 indicate significant differences at 12 h vs. 0 h, ^&&&&^P < 0.0001, ^&&&^P < 0.001, ^&&^P < 0.01, and ^&^P < 0.05 indicate significant differences at 12 h according to repeated-measures ANOVA.) **(D)** Images of H&E-stained ankle tissues in the 4 groups. (Red arrow: inflammatory cell infiltration in the synovial connective tissue. Yellow arrow: pannus in the talus. Green arrow: Synovial connective tissue hyperplasia. Blue arrow: blood vessels in the synovial connective tissue. Purple arrow: inflammatory cell infiltration in the periosteum. Black arrow: Focal necrolysis of connective tissue. Scale bars represent 20 μm for the top row of images and 200 μm for the bottom row of images).

Moxibustion treatments were carried out 3 days before MSU injection. The bilateral Sanyinjiao acupoint (SP 6, located 1 cm straight above the tip of the medial malleolus of the hind limbs) and Zusanli acupoint (ST 36, located 3 mm below the fibular head and lateral to the anterior tubercle of the tibia) were selected as the body stimulation sites for the rats and were shaved for exposure (Xue and Yi, 2021) (Figure 1A). Prior to treatment, the heads of the rats were completely covered with a breathable and opaque headgear to help the rats remain calm during treatment. Moxibustion stimulation at a corresponding temperature of 42°C at the ST36 and SP6 acupoints was applied with a small animal moxibustion strip for 15 min once a day for 6 consecutive days. During the process of moxibustion intervention, a digital temperature recorder was used to detect the temperature of the moxibustion site during, before and after moxibustion, and the target temperature was obtained by adjusting the distance between the moxibustion strips and the acupoint. Rats in the Con group and the AGA group were subjected to the same procedure but were not treated with moxibustion.

Colchicine (0.1 mg/kg) was orally administered once a day for 6 consecutive days 3 days before MSU injection as a positive control (FitzGerald et al., 2020).

### 2.3 Assessment of ankle edema and mechanical allodynia

To determine ankle edema, the diameter of the right ankle was measured before MSU injection and 12, 24, 48, and 72 h after MSU injection (Figure 1B). Mechanical allodynia was assessed using von Frey filaments (Stoelting, Kiel, WI, United States). The right hind paw was stimulated with a series of von Frey filaments (0.4, 0.6, 1.0, 2.0, 4.0, 6.0, 8.0, and 15.0 g). The 50% paw withdrawal threshold (PWT) was determined by using the up-down method as previously described (Lee et al., 2013). The hind paw was stimulated for 3–4 s while the filament was bent several times. Rapid hind paw withdrawal in response to the application of a von Frey filament was considered a positive response. All testing was performed by trained investigators who were blinded to the experimental conditions.

### 2.4 Biological sample collection

All rats were anesthetized with 2.5% tribromoethanol (0.3 mL/100 g body weight) after treatment. 4–6 mL serum samples were collected from the abdominal aorta, and the rats were sacrificed. The ankles of the rats were surgically removed. Three ankles from each group were fixed in 4% paraformaldehyde. The synovium of the other ankles was surgically removed, placed in a 1.5 mL sterilized centrifuge tube, quickly frozen in liquid nitrogen, and then transferred to a −80°C ultralow temperature freezer for storage.

### 2.5 Hematoxylin-eosin and immunohistochemical staining

The dehydrated ankles were decalcified in CalciClear Rapid solution (National Diagnostics, Atlanta, GA, United States) for 15 days, and the samples were embedded in paraffin and sectioned into 5-mm-thick slices with a freezing microtome (CM 1950, Leica Biosystems Division of Leica Microsystems Inc., Germany) for H&E and immunohistochemical staining.

### 2.6 Enzyme-linked immunosorbent assay

IL-18 and IL-1β levels in the serum were detected by using ELISA kits (ED-30204/ED-30206, Jiangsu Meimian Industrial Co., Ltd., China). All procedures were conducted according to the manufacturer’s instructions.

### 2.7 Quantitative real-time PCR

The relative expression of NLRP3, ASC, and Caspase-1 in the synovium was measured by qPCR. Total RNA was extracted from the synovium by the TRIzol method and reverse transcribed into cDNA. Subsequently, cDNA was used as the template for amplification. All the data were analyzed by Quantity One software, and the relative expression levels were calculated according to the 2−^△△^CT method.

### 2.8 Sample preparation and LC-MS analysis

#### 2.8.1 Metabolite extraction

Prior to LC-MS analysis, 100 μL of serum samples was added to a 1.5 mL centrifuge tube with 400 μL of a solution [acetonitrile:methanol = 1:1 (v:v)] containing 0.02 mg/mL internal standard (L-2-chlorophenylalanine) to extract metabolites. The samples were mixed by vortexing for 30 s and low-temperature sonicated for 30 min (5°C, 40 KHz). The samples were placed at −20°C for 30 min to precipitate the proteins. Then, the samples were centrifuged for 15 min (4°C, 13,000 × g). The supernatant was removed, and the sample was blown dry under nitrogen. The sample was then resolubilized with 100 µL of solution (acetonitrile:water = 1:1) and extracted by low-temperature ultrasonication for 5 min (5°C, 40 kHz), followed by centrifugation at 13,000 *g* and 4°C for 10 min. The supernatant was transferred to sample vials for LC-MS/MS analysis.

#### 2.8.2 Quality control sample preparation

As a part of the system conditioning and quality control process, a pooled quality control sample (QC) was prepared by mixing equal volumes of all samples. The QC samples were disposed and tested in the same manner as the analytic samples. It helped to represent the whole sample set, which would be injected at regular intervals (every 6 samples) in order to monitor the stability of the analysis.

#### 2.8.3 LC-MS analysis

LC-MS analysis of the samples was conducted on a Thermo UHPLC-Exploris 240 system equipped with an ACQUITY HSS T3 column (100 mm × 2.1 mm i. d., 1.8 μm; Waters, United States) at Majorbio Bio-Pharm Technology Co., Ltd. (Shanghai, China). The mobile phases consisted of 0.1% formic acid in water: acetonitrile (95:5, v/v) (solvent A) and 0.1% formic acid in acetonitrile:isopropanol:water (47.5:47.5, v/v) (solvent B). The flow rate was 0.40 mL/min, and the column temperature was 40°C. The injection volume was 3 μL.

#### 2.8.4 M conditions

The mass spectrometric data were collected using a Thermo UHPLC-Q Exactive HF-X mass spectrometer equipped with an electrospray ionization (ESI) source operating in positive mode and negative mode. The optimal conditions were as follows: aux gas heating temperature, 350°C; capillary temperature, 320°C; sheath gas flow rate, 60 psi; aux gas flow rate, 20 arb; ion-spray voltage floating (ISVF) voltage, −3500 V in negative mode and 3400 V in positive mode; and normalized collision energy, 20–40–60 eV rolling for MS/MS. The full MS resolution was 60,000, and the MS/MS resolution was 15,000. Data acquisition was performed in Data Dependent Acquisition (DDA) mode. The detection was carried out over a mass range of 70–1,050 m/z.

#### 2.8.5 Data analysis

The raw LC-MS data were converted into a common format by Progenesis QI software (Waters, Milford, United States) through baseline filtering, peak identification, peak integration, retention time correction, and peak alignment. Then, the data matrix containing sample names, m/z values, retention times and peak intensities was exported for further analyses. Moreover, the metabolites were identified by searching databases, and the main databases used were the Human Metabolome Database (HMDB) (http://www.hmdb.ca/), Metlin (https://metlin.scripps.edu/) and the self-compiled Majorbio Database (MJDB) of Majorbio Biotechnology Co., Ltd. (Shanghai, China).

The data matrix obtained by searching the database was uploaded to the Majorbio cloud platform (https://cloud.majorbio.com) for data analysis. First, the data matrix was preprocessed as follows. At least 80% of the metabolic features detected in any set of samples were retained. After filtering, the minimum value in the data matrix was selected to fill the missing value, and each metabolic signature was normalized to the sum. To reduce the errors caused by sample preparation and instrument instability, the response intensities of the sample MS peaks were normalized using the sum normalization method to obtain the normalized data matrix. Moreover, the variables of QC samples with a relative standard deviation (RSD) > 30% were excluded and log10-transformed to obtain the final data matrix for subsequent analysis.

Then, the R package “ropls” (version 1.6.2) was used to perform principal component analysis (PCA), least partial squares discriminant analysis (PLS-DA), and 7-cycle interactive validation to evaluate the stability of the model. PLS-DA, a supervised discriminant analysis method, is a multivariate statistical analysis method. The method utilizes partial least squares regression to establish a regression model between metabolite expression and sample category to achieve the prediction of sample category. The PLS-DA model of each comparison group was established, and the model evaluation parameters R2Y (model interpretability) and Q2 (model predictability) were obtained by cross-validation, and if R2Y and Q2 were closer to 1, it indicated that the model was more stable and reliable. PLS-DA score plots are often used to visualize the classification effect of the model, and the greater the separation of the two groups of samples in the plot, the more significant the classification effect. The metabolites with VIP > 1 and p < 0.05 were determined to be significantly different based on the variable importance in projection (VIP) obtained by the PLS-DA model and the p value generated by Student’s t-test.

Differentially abundant metabolites among the two groups were mapped to their biochemical pathways through metabolic enrichment and pathway analysis based on the Kyoto Encyclopedia of Genes and Genomes (KEGG) database (http://www.genome.jp/kegg/). These metabolites can be classified according to the pathways they are involved in or the functions they perform. The Python package “scipy.stats” (https://docs.scipy.org/doc/scipy/) was used to perform enrichment analyses and obtain the most relevant biological pathways for the experimental treatments.

#### 2.8.6 Univariate analysis

All of the data are expressed as the means ± SDs, and graphs were generated using GraphPad Prism 4.0 software (Hearne Scientific Software). The differences in the concentrations of metabolites between the two groups were analyzed using independent-sample t tests with SPSS 19.0 software. Additionally, one-way ANOVA with Tukey’s multiple comparisons *post hoc* test in SPSS 19.0 software was applied to verify the differences in the ankle diameter,PWT and IL-18, IL-1β,Caspase-1, NLRP3, and ASC expression among the four groups. *P* value < 0.05 indicated a statistically significant difference.

## 3 Results

### 3.1 Effects of moxibustion treatment on MSU-induced GA model rats

In a preliminary study, we measured ipsilateral ankle edema and mechanical allodynia before MSU injection, and 12, 24, 48, 72 h after MSU injection (n = 8 in each group). Visually, the hind paw injected with MSU was swollen, and ankle edema increased. After 72 h, as the swelling subsided, the experimental animals became more active. The next experiment was conducted in the same way as the preliminary study, and edema and mechanical allodynia were evaluated at 72 h after MSU injection. To evaluate the effects of moxibustion and colchicine in a gouty animal model, we evaluated ankle edema in the rats. In the MSU-injected group, there was a sharp increase in ankle edema at 12 h after injection, which became more severe at 48 h after injection then decreased gradually from 48 h to 72 h. In the case of the Mox-treated and the Col-treated groups, the initial 24 h showed a similar pattern to that of the model group; the ankle edema of the Mox-treated group decreased significantly after 24 h, but that of the Col-treated group did not decrease significantly until after 48 h. These results suggest that moxibustion and colchicine mitigate MSU crystal-induced ankle edema. The effect was greater in the moxibustion group than in the colchicum group (Figure 1B).

The PWT was measured 12, 24, 48, and 72 h after MSU injection using the von Frey test. Compared to that before MSU injection, the PWT at 12 h decreased in all of the groups. The PWT increased significantly after 24 h in the control group, and that of the AGA, Mox and Col groups increased significantly after 48 h. The PWT in the Mox and Col groups increased significantly compared with that of the AGA group after 48 and 72 h (Figure 1C).

Pathological changes in the gastric tissues of the various groups were evaluated by HE staining (Figure 1D). Compared with the Con group tissue, the AGA group tissue showed a small amount of necrotic tissue fragments and eosinophils in the joint cavity and a small area of pannus (yellow arrow) in the talus. Moreover, there was a small amount of connective tissue hyperplasia and erosion to the cartilage. There were many new blood vessels (blue arrow) in the synovial connective tissue, and there were many lymphocytes, granulocytes and macrophages in the synovial connective tissue (red arrow) and periosteum (purple arrow). However, after moxibustion or colchicine intervention, the cartilage surface became smooth, the number of chondrocytes became abundant, and the infiltration of lymphocytes, granulocytes, macrophages, lymphocytes and macrophages decreased significantly. The histological evaluation indicated the powerful gastroprotective effect of moxibustion and colchicine on GA model rats.

**FIGURE 2:**
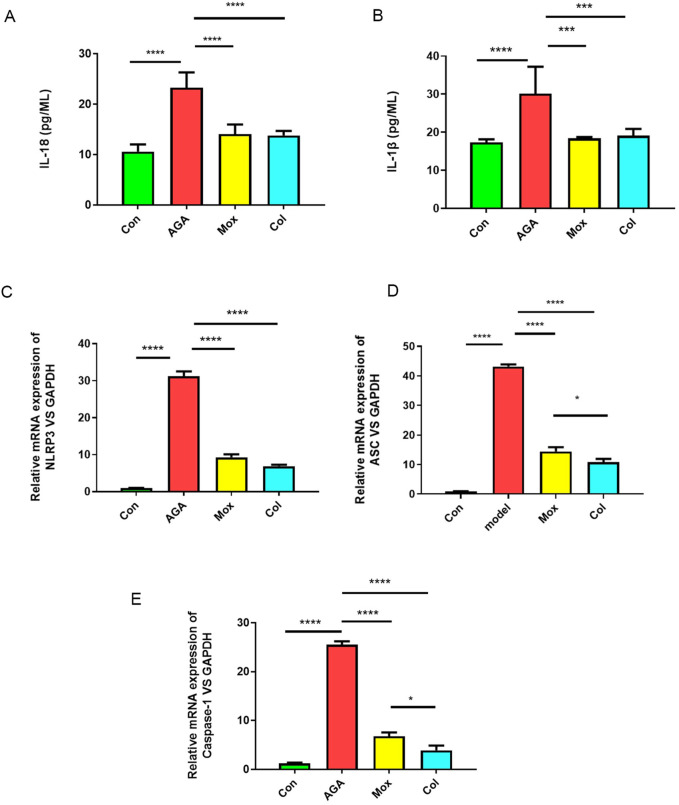
Effects of moxibustion or colchicine on proinflammatory cytokines and genes in gouty rats. **(A, B)** The serum levels of proinflammatory cytokines (IL-18 and IL-1β) in the 4 groups were measured by ELISA (n = 6/group). **(C**–**E)** qPCR analysis of proinflammatory genes (NLRP3, ASC, and Caspase-1) in the 4 groups. The data are presented as the means ± SDs. ****P < 0.0001, ***P < 0.001, **P < 0.01, *P < 0.05 indicate significant differences vs the AGA group. Con: control, AGA: acute gouty arthritis model, Mox: moxibustion, Col: colchicine.

Compared with those in the control group, the serum levels of IL-18 and IL-1β in the AGA group increased (P < 0.05). Compared with those in the AGA group, the serum levels of IL-18 and IL-1β in the Mox and Col groups decreased (P < 0.05) (Figures 2A, B).

To determine the effect of moxibustion or colchicine on the ankle synovial membrane, the relative expression of NLRP3, ASC, and Caspase-1 was assessed. The results showed that the expression of NLRP3, ASC, and Caspase-1 was higher in the AGA model group than in the control group (P < 0.05). We found that the levels of NLRP3, ASC, and Caspase-1 decreased after Mox treatment or Col treatment (Figures 2C–E).

**FIGURE 3:**
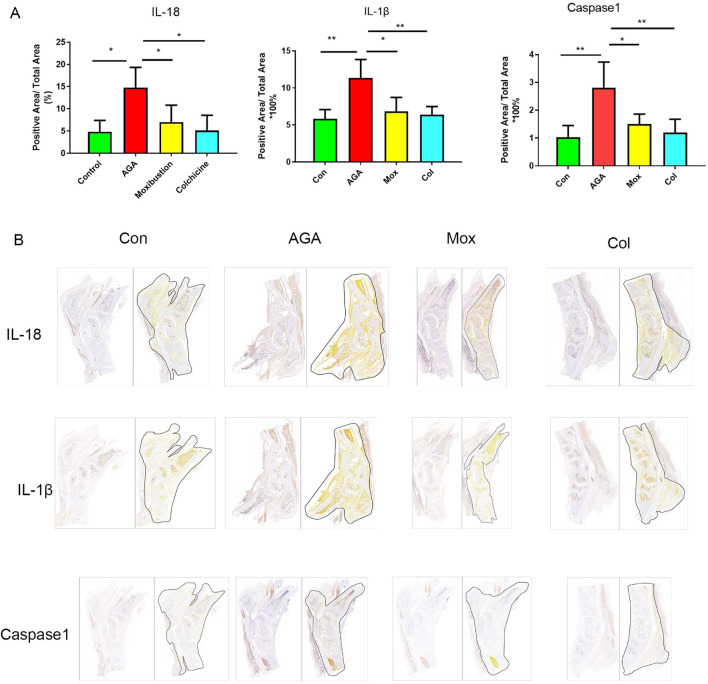
Immunohistochemistry analysis of IL-18, IL-1β and Caspase-1 levels in synovial tissue from the ankle. **(A)** Representative images of positive IL-18, IL-1β and caspase-1 expression in the ankle tissues of the 4 groups. **(B)** Quantification of the expression of IL-18, IL-1β and caspase-1 in the ankle. Con: control, AGA: acute gouty arthritis model, Mox: moxibustion, Col: colchicine.

Immunohistochemical analyses revealed the levels of IL-18, IL-1β and Caspase-1 (Figures 3A, B) in the ankle tissues of the four groups. The levels of IL-18, IL-1β and Caspase-1 in the ankle tissues of rats in the AGA group were greater than those in the ankle tissues of rats in the Con group, which was consistent with previous studies showing that mitochondrial damage after macrophages phagocytose MSU crystals stimulates the expression of related proinflammatory factors. Moreover, the levels of IL-18, IL-1β and Caspase-1 in the ankle tissues of rats in the Mox and Col groups were lower than those in the ankle tissues of rats in the AGA group.

### 3.2 LC-MS spectra and recognition analysis

LC-MS spectra and recognition analysis revealed 15,325 peaks in the 24 serum samples from the 4 groups; 2,015 identified metabolites were extracted, and 1,032 metabolites were annotated in the KEGG database (Supplementary Figures S2A–D; Supplementary Tables S1–S4). The cumulative abundance of MS peaks before preprocessing was 75.33%, and after preprocessing, it was 79.72%, both of which were greater than 70%. For the overall data, the relative standard deviation (RSD, or coefficient of variation (CV) of the QC sample was <0.3, and the cumulative abundance of peaks was >70%, indicating that the data were qualified (Supplementary Figure S2E).

**FIGURE 4:**
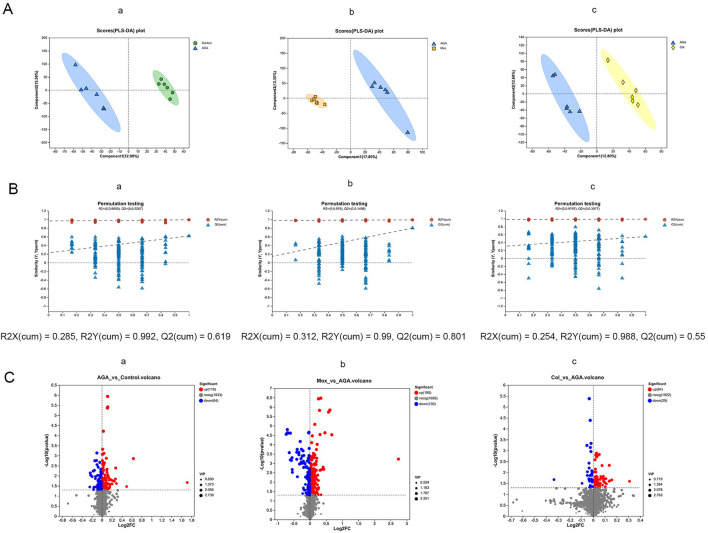
PLS-DA scoring plots **(A)**, permutation test plot plots **(B)** and volcano plots **(C)** for the LC-MS spectra recorded of serum samples from the Con (n = 6), AGA (n = 6), Mox (n = 6), and Col (n = 6) groups of SD rats. (First row of images, AGA vs. Con; second row of images, Mox vs. AGA; third row of images, Col. vs. AGA. The corresponding validation plots were obtained by using the 200-permutation test.). Con, control, AGA, acute gouty arthritis model, Mox, moxibustion, Col: colchicine.

For an overview of the metabolic profiles of the four groups, unsupervised PCA with two principal components was applied. The four groups clustered into distinct groups (Supplementary Figure S1). Next, supervised PLS-DA scoring plots were constructed to describe the separation and comparison among multiple groups of serum samples. The robustness of the PLS-DA models was confirmed by the 200-permutation test,as shown in Figures 4A, B. A clear separation between the Con and AGA groups was observed, indicating that the serum metabolic pattern was significantly changed by AGA modeling. Moreover, a clear separation of the Mox (Figure 4) and Col groups (Figure 4) from the AGA group was observed in the PLS-DA plots.

### 3.3 Differentially abundant metabolites among the 4 groups

Based on the metabolites with VIP values exceeding 1 obtained from the PLS-DA models, we performed t tests and constructed a volcano plot to identify the characteristic metabolites distinguishing the AGA vs Con, Mox vs. AGA, and Col vs. AGA groups (Figure 4C).

The AGA group exhibited 182 characteristic serum metabolites relative to the Con group. Among these characteristic metabolites, the abundance of 118 was increased and the abundance of 64 was reduced compared with those in the Con group (Figures 4C-a). In contrast, the Mox group had 320 characteristic serum metabolites. Among these characteristic metabolites, the abundance of 190 was elevated and the abundance of 130 was reduced compared with those in the AGA group (Figures 4C-b). Moreover, the Col group had 93 characteristic serum metabolites. Similarly, the abundance of 64 and 29 of these characteristic metabolites was elevated and reduced, respectively, compared to those in the AGA group (Figure 4C-c).

Overall, our research indicated that the application of moxibustion and colchicine had regulatory effects on the serum metabolic profile of AGA rats. This is evidenced by the distinct changes in the abundance of various metabolites observed in response to Mox and Col treatment.

As shown in Table 1, quantitative comparison of characteristic serum metabolites among the Con, AGA and Mox groups of rats based on relative LC‒MS analysis revealed that there were 351 significantly differentially abundant metabolites, and only 62 metabolites had VIP (PLS-DA) values > 1 in the Con vs. AGA and Mox vs. AGA groups. Moreover, quantitative comparison of the abundance of characteristic serum metabolites among the Con, AGA and Col groups of rats based on LC‒MS data revealed 262 significantly differentially abundant metabolites, 11 of which had VIP (PLS-DA) value > 1 in the Con vs. AGA and Col vs. AGA groups and were among the characteristic serum metabolites among the Con, AGA and Col groups of rats.

**TABLE 1:** Quantitative comparison of characteristic metabolite levels of plasma among Con, AGA, Mox, and Col group of rats based on relative NMR integrals.

Metabolitc		Mean ± SD				VIP score		
Num	Metabolite name	Con (N = 6)	AGA (N = 6)	Mox (N = 6)	Col (N = 6)	VIP1	VIP2	VIP3
1	7-Ketodeoxycholic acid	6.352 ± 0.1555*	6.623 ± 0.1618	6.253 ± 0.2274*	6.336 ± 0.1781*	1.95	1.75	1.98
2	(5E)-3-Hydroxyhept-5-enoylcarnitine	4.888 ± 0.02741*	4.93 ± 0.02508	3.77 ± 0.01655**	5.019 ± 0.01931***	1.88	1.89	2.67
3	L-DOPA n-Butyl Ester	4.844 ± 0.02052	4.876 ± 0.02295	4.276 ± 0.03654**	4.919 ± 0.02292*	1.79	1.83	2.11
4	N-methylsphingosine	4.672 ± 0.03375*	4.722 ± 0.02006	4.264 ± 0.04025*	4.785 ± 0.02247**	2.02	1.75	2.49
5	Imidazolone A	4.532 ± 0.03185*	4.582 ± 0.01879	4.197 ± 0.1819***	4.691 ± 0.023***	2.07	2.06	2.76
6	(4E)-3-Hydroxyhex-4-enoylcarnitine	4.495 ± 0.0264**	4.553 ± 0.03221	4.461 ± 0.02092***	4.63 ± 0.02237***	2.10	2.04	2.45
7	Threonic Acid	4.551 ± 0.01585**	4.51 ± 0.01223	4.638 ± 0.0256***	4.463 ± 0.02642**	2.43	2.06	2.28
8	Imidazoleacetic acid riboside	4.465 ± 0.03228	4.606 ± 0.07743	4.677 ± 0.02525***	4.426 ± 0.1615*	2.27	2.24	1.80
9	Hypoxanthine	4.37 ± 0.02537	4.334 ± 0.01422	4.794 ± 0.02218***	4.271 ± 0.03322**	1.97	2.14	2.35
10	3-Oxoheptanoylcarnitine	4.082 ± 0.07171*	4.175 ± 0.05318	4.94 ± 0.03173**	4.25 ± 0.04305	1.80	1.91	1.89
11	M-Xylene	3.695 ± 0.02037	3.726 ± 0.02257	5.02 ± 0.03414***	3.763 ± 0.01922*	1.77	2.08	2.09
12	P-CHLOROPHENYLALANINE	8.241 ± 0.01711	8.265 ± 0.02012	8.296 ± 0.01947*		1.69	1.60	
13	PE (15:0/20:0)	6.402 ± 0.03414*	6.318 ± 0.05947	6.386 ± 0.03893		1.97	1.52	
14	Triptohypol F	6.379 ± 0.08669*	6.247 ± 0.06414	6.372 ± 0.04963*		1.97	1.87	
15	Demissidine	5.971 ± 0.06311	5.845 ± 0.05962	6.061 ± 0.1292**		2.14	1.85	
16	4beta-hydroxymethyl-4alpha-methyl-5alpha-cholest-7-en-3beta-ol	5.941 ± 0.09607	5.831 ± 0.07241	5.965 ± 0.04847*		1.65	1.86	
17	3-Dehydrocholic Acid	5.715 ± 0.2095	5.938 ± 0.1142	5.634 ± 0.2082*		1.68	1.71	
18	2-Mercaptobenzothiazole	5.701 ± 0.04381*	5.629 ± 0.02915	5.705 ± 0.03416**		2.08	1.93	
19	N-Nervonoyl Asparagine	5.549 ± 0.2519	5.234 ± 0.1476	6.172 ± 0.4171***		1.84	2.07	
20	24-Methylcycloart-23-en-3beta-yl acetate	5.623 ± 0.0762*	5.487 ± 0.03839	5.665 ± 0.1049**		2.23	1.89	
21	PC(20:1 (11Z)/20:4 (8Z,11Z,14Z,17Z))	5.556 ± 0.04941	5.497 ± 0.02585	5.398 ± 0.08621*		1.80	1.58	
22	FAHFA (22:6 (4Z,7Z,10Z,13Z,16Z,19Z)/13-O-18:2 (9Z,11 E))	5.331 ± 0.2747	5.004 ± 0.1137	5.763 ± 0.3746**		1.86	2.02	
23	4-Nonylphenol	5.36 ± 0.1142	5.202 ± 0.09608	5.474 ± 0.1255**		1.82	1.94	
24	Alpha-Bisabolol oxide B	5.339 ± 0.1069	5.212 ± 0.0883	5.373 ± 0.04798*		1.65	1.90	
25	Cedrol	5.351 ± 0.1203*	5.206 ± 0.06101	5.338 ± 0.08004		1.83	1.74	
26	Bredemolic acid	5.24 ± 0.1098	5.039 ± 0.1243	5.598 ± 0.2738***		1.96	1.99	
27	Carpaine	5.212 ± 0.1465	4.979 ± 0.1231	5.675 ± 0.3016***		1.97	2.08	
28	Hydroxyphenylacetylglycine	5.236 ± 0.5275	4.548 ± 0.3613	6.028 ± 0.5492***		1.83	2.11	
29	Ribavirin monophosphate	5.271 ± 0.01455**	5.24 ± 0.01114	5.269 ± 0.01395**		2.29	1.90	
30	Cis-Uvariamicin IB	5.244 ± 0.4168*	4.416 ± 0.5896	5.851 ± 0.4054***		1.90	2.04	
31	2-Hexadec-7-enylicosa-8,11-dienedioic acid	5.099 ± 0.2864	4.738 ± 0.1664	5.604 ± 0.4318***		1.85	2.00	
32	FAHFA (22:6 (4Z,7Z,10Z,13Z,16Z,19Z)/14-O-22:6 (4Z,7Z,10Z,13Z,16Z,19Z))	5.097 ± 0.3431	4.64 ± 0.3362	5.601 ± 0.3739**		1.70	2.01	
33	Glycyrrhetinic Acid	4.99 ± 0.1707	4.756 ± 0.1778	5.414 ± 0.2954***		1.70	2.01	
34	Maslinic acid	4.935 ± 0.1869	4.693 ± 0.1675	5.47 ± 0.3671***		1.72	2.02	
35	Tsugaric acid A	4.971 ± 0.3183	4.452 ± 0.3685	5.639 ± 0.4059***		1.82	2.08	
36	Exo,exo-1,8-Epoxy-p-menthane-2,6-diol	4.852 ± 0.11	4.978 ± 0.02837	4.87 ± 0.0816		1.87	1.69	
37	Chenodeoxycholylglycine	4.634 ± 0.39	5.241 ± 0.1719	4.696 ± 0.5671		2.12	1.41	
38	2-Quinolinecarboxylic acid	4.718 ± 0.1045**	5.098 ± 0.1934	4.692 ± 0.1411**		2.29	1.96	
39	5-Methoxyindoleacetate	4.764 ± 0.1796*	5.045 ± 0.1648	4.627 ± 0.1861**		1.91	1.94	
40	Vitamin K1	4.704 ± 0.2208	4.331 ± 0.2686	5.239 ± 0.358***		1.83	2.05	
41	Dichlorophenyl-bis-triazolylpropanol	4.8 ± 0.09961***	5.156 ± 0.1523	4.285 ± 0.09945***		2.39	2.35	
42	Dehydrocarpaine I	4.603 ± 0.1634	4.339 ± 0.1597	5.021 ± 0.2489***		1.91	2.12	
43	3-methoxy Limaprost	4.68 ± 0.1002	4.55 ± 0.06725	4.683 ± 0.1086		1.83	1.53	
44	Pregnanediol 3-O-glucuronide	4.441 ± 0.2667*	4.861 ± 0.1933	4.394 ± 0.3091*		2.01	1.71	
45	Acetoxolone	4.522 ± 0.07441	4.369 ± 0.05479	4.715 ± 0.1879***		2.26	1.96	
46	4-Hydroxy-3-methoxy-cinnamoylglycine	4.49 ± 0.05485	4.596 ± 0.09208	4.362 ± 0.1561**		1.74	1.73	
47	Penicillic Acid	4.371 ± 0.2629	3.999 ± 0.1305	5.066 ± 0.4562***		2.01	2.11	
48	Tetracos-15-enoic acid	4.382 ± 0.1415	4.697 ± 0.1366	4.257 ± 0.45		2.23	1.43	
49	(R)-2-Benzylsuccinate	4.557 ± 0.1378	4.703 ± 0.07667	3.921 ± 0.5787**		1.67	1.75	
50	Misonidazole	4.373 ± 0.0236*	4.329 ± 0.02958	4.379 ± 0.02572*		1.90	1.70	
51	Kainic acid	4.291 ± 0.2026	4.582 ± 0.2103	4.161 ± 0.1741**		1.75	1.89	
52	Belinostat glucuronide	4.237 ± 0.1469	4.707 ± 0.2897	3.542 ± 0.5996***		2.14	1.95	
53	N-Acetyl-L-phenylalanine	3.996 ± 0.09628	4.145 ± 0.06158	4.003 ± 0.1388		2.03	1.43	
54	Floctafenic acid	4.056 ± 0.01419*	4.009 ± 0.0274	4.056 ± 0.03622*		2.17	1.50	
55	Scopoletin	3.824 ± 0.6238	3.125 ± 0.161	4.865 ± 0.7608***		1.84	2.10	
56	Indican	3.808 ± 0.09673	3.977 ± 0.1215	3.697 ± 0.174**		1.86	1.75	
57	Dehydrocyanaropicrin	4.22 ± 0.5925***	2.721 ± 0.5953	4.522 ± 0.1318***		2.32	2.23	
58	(2-Hydroxyethoxy)acetic acid	3.74 ± 0.03791*	3.809 ± 0.03136	3.888 ± 0.04469*		2.11	1.82	
59	Seryltryptophan	3.605 ± 0.2069	3.983 ± 0.07317	3.476 ± 0.4533*		2.29	1.61	
60	Beta-D-ribosylnicotinate	3.609 ± 0.5086	2.993 ± 0.02609	4.453 ± 0.7411***		1.96	2.03	
61	Methoxyphenylacetic acid	4.051 ± 0.2316	3.778 ± 0.1765	3.148 ± 0.5523*		1.69	1.56	
62	(+)-Bornyl diphosphate	3.319 ± 0.295**	3.888 ± 0.3183	2.604 ± 0.02856***		2.04	2.32	

Note: (1) Statistical significances: *P < 0.05; **P < 0.01, ***P < 0.001 vs. AGA group by t-test. (2) VIP1, VIP2 and VIP3 represent variable importance in the projection (VIP) scores of metabolites for pair-wise comparisons of AGA vs. Con, Mox vs. AGA, and Col vs. AGA from the PLS-DA models, respectively.

**FIGURE 5:**
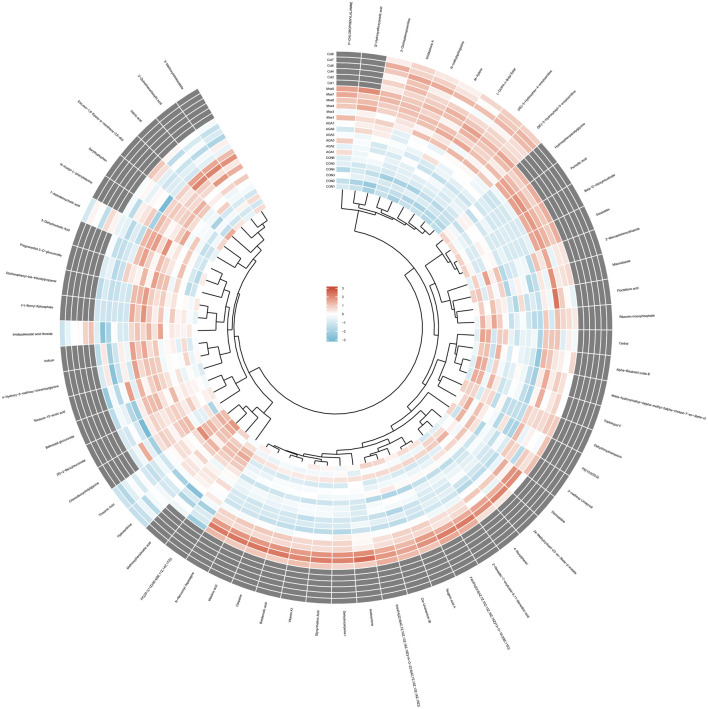
Heatmap metabonomic data depicting the data structure of 62 biomarkers. The depth of the color represents the size of the variable.

To visually analyze the metabolite changes *in vivo*, we used hierarchical cluster analysis (i.e., heatmap) to analyze the biomarkers. The heatmap visually presented 62 distinct diagnostic biomarkers in all four groups (Figure 5). Showed the variable information as the ordinate and the sample information as the abscissa, and the depth of the color represents the size of the variable. A closer bifurcation of the variable information in the vertical axis indicated greater similarity between substances, suggesting that they were probably derived from metabolites of the same substance.

### 3.4 Pathway enrichment analysis revealed significantly altered metabolic pathways among the 4 groups

**FIGURE 6:**
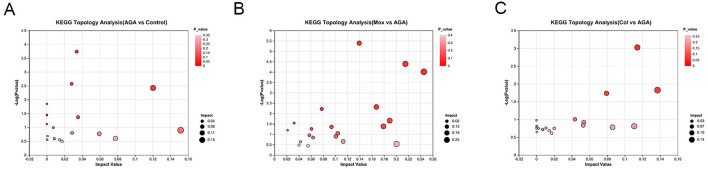
Topological analysis of the significantly altered metabolic pathways among the AGA, Mox and Col groups. **(A)** The significantly different metabolic pathways between the AGA and Con groups. **(B)** The significantly different metabolic pathways between the Mox and AGA groups. **(C)** The significantly altered pathways between the Col and AGA groups. Con: control, AGA: acute gouty arthritis model, Mox: moxibustion, Col: colchicine.

Using a combination of metabolite set enrichment analysis (Figure 6) (P < 0.05) and pathway impact value (PIV ≥ 0.05), we identified 1 significantly altered metabolic pathway in the AGA group, 8 significantly altered metabolic pathways in the Mox group and 3 significantly altered metabolic pathways in the Col group compared to the AGA group. Among these pathways, the Mox, Col and AGA groups shared 1 significantly altered metabolic pathway, while the Mox and Col groups shared 2 significantly altered metabolic pathways (Table 1). In addition, the Col group exhibited a unique pathway, namely, nucleotide metabolism, while the Mox group exhibited six unique pathways: the tricarboxylic acid (TCA) cycle; pantothenate and CoA biosynthesis; alanine, aspartate and glutamate metabolism; glyoxylate and dicarboxylate metabolism; pyruvate metabolism; and tyrosine metabolism. Overall, there were more significantly altered metabolic pathways identified in the Mox group than in the Col group (Table 2).

**TABLE 2:** Significantly altered metabolic pathways identified in AGA vs. Con, and Mox, Col vs. AGA in GA rats.

Num Pathway desciption		AGA vs Con	Mox vs AGA	Col vs AGA
1	Nicotinate and nicotinamide metabolism		√	√
2	Glycerophospholipid metabolism	√	√	√
3	Nucleotide metabolism			√
4	Citrate cycle (TCA cycle)		√	
5	Pantothenate and CoA biosynthesis		√	
6	Alanine, aspartate and glutamate metabolism		√	
7	Glyoxylate and dicarboxylate metabolism		√	
8	Pyruvate metabolism		√	
9	Tyrosine metabolism		√	

## 4 Discussion

This study demonstrated the therapeutic effects and mechanism of moxibustion treatment on AGA. First, moxibustion and colchicine relieves edema and mechanical allodynia by reducing inflammation in the ankle and decreasing the levels of proinflammatory cytokines in MSU-induced GA model rats. Second, both moxibustion and colchicine treatment restored the serum metabolic profile of GA model rats.

In the present study, both moxibustion and colchicine improved gait, joint swelling and pain thresholds in AGA rats, but moxibustion promoted the recovery of joint swelling significantly earlier than colchicine, which may be related to the pretreatment of the moxibustion group. This finding provides ideas for the subsequent development of a study on the quantitative efficacy of moxibustion, as well as a study on the timing of moxibustion intervention.

Inflammasomes are cytoplasmic high-molecular-weight protein complexes that activate Caspase-1 in response to microbial invasion and injury signals. They consist of a nucleotide-binding oligomerization domain (NOD)-like receptor (NLR), an apoptosis-associated speck-like protein containing a caspase recruitment domain (CARD) (ASC), and the effector protease Caspase-1 (Tschopp and Schroder, 2010). The NLRP3 inflammasome belongs to a family of inflammasomes that are responsible for activating inflammatory responses in response to specific cellular stimuli. ASC is the bridge between NLRP3 and Caspase-1. During inflammasome assembly, NLRP3 interacts with the N-terminal end of the junction protein ASC through a PYD-PYD interaction. Moreover, the C-terminal end of ASC has a CARD, which can bind to procaspase-1 through a CARD-CARD interaction to promote caspase dimerization and activation and thus participate in the proinflammatory response (McKee and Coll, 2020; Kelley et al., 2019). The activation of the NLRP3 inflammasome triggers the transformation of pro-IL-1β and pro-IL-18 into their biologically active forms, as well as in the cleavage of gasdermin D (GSDMD), which promotes pyroptosis (Guo et al., 2015). Moreover, the proinflammatory IL-18 and IL-1β can be released by macrophages (Mezzasoma et al., 2023). After monosodium urate (MSU) is deposited in the joint cavity, it is phagocytosed by macrophages, which induces cellular mitochondrial damage and generates reactive oxygen species (ROS), which in turn activate the NLRP3 inflammasome signaling pathway, leading to the maturation and release of IL-18, IL-1β and other proinflammatory factors (Chen et al., 2019). NLRP3, ASC, and Caspase-1 were downregulated in the Mox group and Col group, indicating that moxibustion and colchicine have similar effects on inhibiting joint inflammation.

The metabolomics analysis revealed that moxibustion and Colchicine treatment could effectively ameliorate the aberrant metabolic patterns of AGA rats. There were common regulatory pathways involved in the metabolic pathway by which moxibustion and colchicine reduce joint inflammation in AGA rats, while different metabolic control pathways also existed. Overall, moxibustion had significant effects on 62 key metabolites in the serum and 8 pathways, while colchicine had significant effects on 11 key metabolites in the serum and 2 pathways. Therefore, it can be speculated that moxibustion may regulate the metabolism of AGA rats more extensively than colchicine. This finding is consistent with previous research suggesting that acupuncture transmits signals and regulates various biochemicals in the body through exogenous stimulation, which involves multiple targets, processes and pathways (Chen et al., 2016). Although there are two common metabolic pathways, glycerophospholipid metabolism and nicotinate and nicotinamide metabolism, the two treatments may act on different targets, so focusing on specific changes in metabolites can explain the underlying mechanism, as described below.

### 4.1 Glycerophospholipid metabolism

Glycerophospholipids are components of bile and cell membranes that are involved in biofilm formation, cell membrane recognition, and signaling and can ultimately be hydrolyzed into free fatty acids and lysophospholipids (Iliadi et al., 2018). One study revealed significant differences in glycerophospholipid metabolism between gout patients and controls via an metabolomic analysis of urine specimens (Li et al., 2018). An increasing number of animal experiments have identified lipid metabolism disorders in hyperuricemic or gouty rats, with the glycerophospholipid metabolic pathway being the most affected pathway (Yang et al., 2021; Lyu et al., 2019). It has been suggested that the glycerophospholipid metabolic pathway is a common pathway for moxibustion and colchicine intervention in AGA rats, which is consistent with the findings of the presence of metabolites related to glycerophospholipid metabolism among the serum metabolites of patients with gout; therefore, the glycerophospholipid metabolic pathway could be the core pathway for the treatment of gouty arthritis. However, the metabolic targets of moxibustion and colchicine are somewhat different.

### 4.2 Nicotinate and nicotinamide metabolism

Nicotinate and nicotinamide metabolism refers to the metabolism of nicotinate (vitamin B3 or nicotinamide) and its derivative nicotinamide (nicotinic acid or nicotinamide) in the human body. Nicotinate is present in the form of nicotinamide in animals and plants, but it is absorbed in the intestine in the form of nicotinic acid after deamination. It is used in the human body to synthesize nicotinamide adenine dinucleotide (NAD). Subsequently, in nicotinamide metabolism, nicotinamide is released from NAD through poly (AD ribosyl), which is methylated in the liver and excreted into the urine in the form of MNA (1-methyl nicotinamide) (Fukushima, 2005; Periyasamy et al., 2019). NAD is involved in a wide range of processes, including the regulation of the cellular redox state, energy metabolism and mitochondrial biogenesis (Tannous et al., 2021). In addition, NAD acts as a signaling molecule and is a cosubstrate for a variety of enzymes, such as deacetylases and phosphoribosyltransferases, and some extracellular enzymes (such as CD38), regulating key biological processes such as gene expression, DNA repair, calcium signaling and circadian rhythm (Satoh et al., 2021). Uric acid is the final product of purine metabolism, or the degradation of adenine and guanine. Phosphoribosyltransferase (PRPP synthetase) plays an important role in regulating purine metabolism (Jakse et al., 2019; Gherghina et al., 2022). Moreover, it is well known that uric acid metabolism is a determining factor in gout regression (Shah and Keenan, 2010). The abundance of N1-Methyl-2-pyridone-5-carboxamide and beta-D-ribosylnicotinate was significantly increased in the AGA group, and in the Mox group, the abundance of succinic acid semialdehyde was significantly increased, while the abundance of beta-D-ribosylnicotinate and nicotinic acid was significantly decreased, and the abundance of succinic acid semialdehyde and niacinamide was significantly increased in the Col group. Succinic acid semialdehyde is an intermediate product of the fatty acid metabolism pathway. In the process of fatty acid β-oxidation, fatty acids are broken down into succinic acid semialdehyde and acetyl coenzyme A. Succinic acid semialdehyde further undergoes decarboxylation to generate acetyl coenzyme A and thus participates in the TCA cycle. Therefore, moxibustion and colchicine can correct the metabolic disorders of AGA by increasing nicotinamide and nicotinamide metabolism.

### 4.3 The TCA cycle

When the body’s energy decreases due to TCA cycle disorder, a hypoxic and anoxic environment develops locally in the joints, leading aberrant adenosine triphosphate (ATP) production and exacerbating the destruction of articular bone (Du et al., 2017; Guo et al., 2021). Gouty arthritis leads to increased mitochondrial dysfunction and results in increased mitochondrial ROS production, promoting inflammatory cell activation (Mezzasoma et al., 2023; Chen et al., 2019). Previous studies have shown that acupuncture can regulate the TCA cycle. A study has found that the increased plasma concentration of saccharides and decreased lactate concentration in healthy young men after acupuncture at the Zusanli, Liangmen and Jvliao acupoints were associated with TCA energy metabolism (Wu et al., 2010). Hot tonic acupuncture could improve rheumatoid arthritis of rabbit regulating the TCA cycle and glucose metabolism (Du et al., 2017). In the present study, moxibustion was found to increase the abundance of aconitate, oxaloacetate (Hernandez-Baixauli et al., 2022), isocitrate, isocitrate, and malic acid in AGA rats, and Hernandez’s study confirmed a decrease in metabolism associated with TCA in a rat model of chronic inflammation, which was manifested by a significant decrease in the levels of its intermediates aconitate, isocitrate, and malic acid indicating that moxibustion can play an anti-inflammatory role by restoring TCA energy metabolism.

One limitation of this study is that only one moxibustion therapy was used. Different moxibustion treatments will be included in future studies to further explore the therapeutic effects of moxibustion on GA model rats and its potential mechanisms.

## 5 Conclusion

In conclusion, we found that moxibustion and colchicine have similar effects on inhibiting joint inflammation through downregulating the NLRP3, ASC, and Caspase-1 related signal pathway. Moxibustion and colchicine can restore the metabolism of glycerol phospholipids, nicotinic acid and nicotinamide in AGA rats. Compared with colchicine, moxibustion may regulate the metabolism of AGA rats more broadly, especially in terms of energy metabolism. Our research deepens our understanding of the complex mechanisms of moxibustion and promotes the application of different methods in the clinical practice.

## Data Availability

The original contributions presented in the study are publicly available. This data can be found here: MetaboLights repository, accession number MTBLS12225.
