# Supplementary material for: Moxibustion inhibits inflammation in monosodium urate crystal-induced gouty arthritis model rats through metabolomic regulation
Source: Front Mol Biosci. 2025 Mar 3;12:1433912. doi: 10.3389/fmolb.2025.1433912 (PMC11911207; doi:10.3389/fmolb.2025.1433912)
Supplement: Supplementary file 4 [file Image2.pdf]

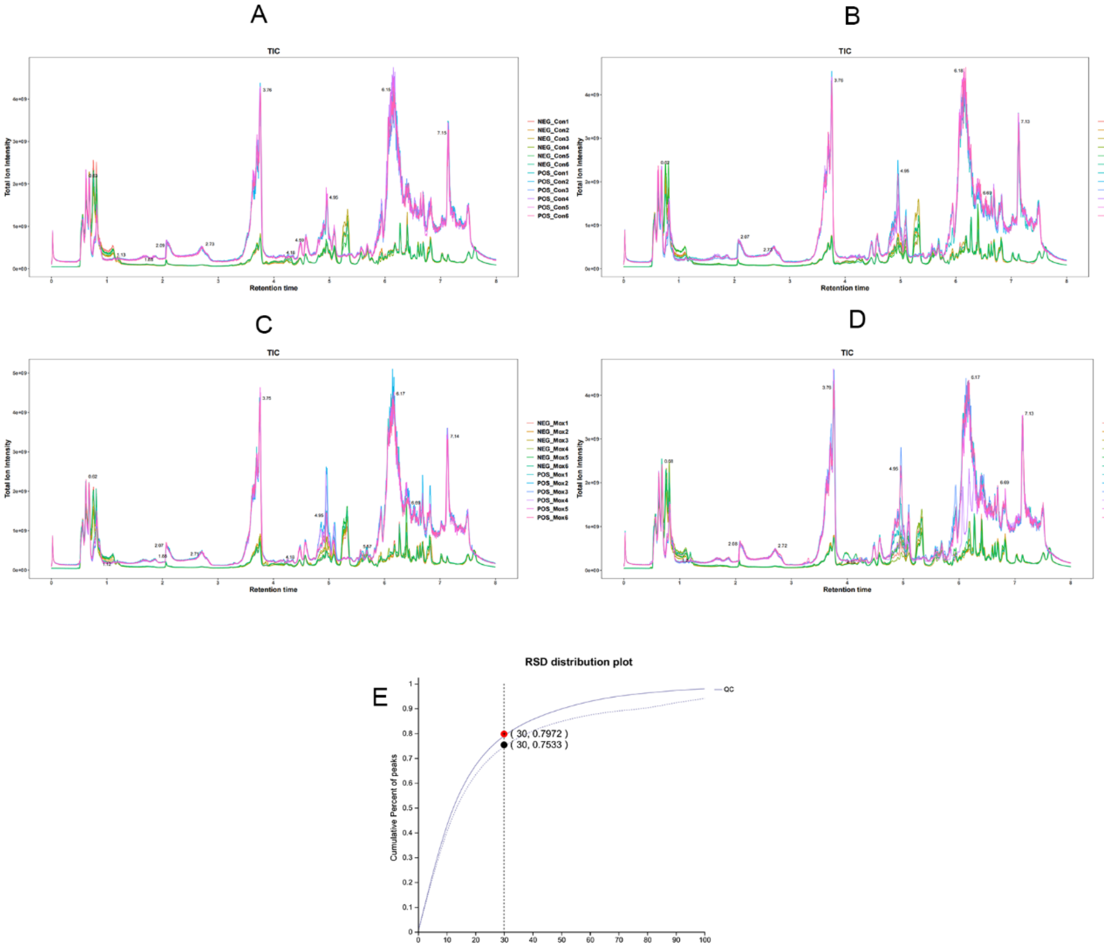

Fig. S1.LC–MS spectra of serum extracts from the 4 groups. (A) Con group, (B) AGA group, (C) Mox group, (D) Col group (E) Original data sample evaluation chart. The dotted line represents before preprocessing and the solid line represents after preprocessing. Con: control, AGA: acute gouty arthritis model, Mox: moxibustion, Col: colchicine.
