# Supplementary figures and images for: Moxibustion inhibits inflammation in monosodium urate crystal-induced gouty arthritis model rats through metabolomic regulation
Source: Front Mol Biosci. 2025 Mar 3;12:1433912. doi: 10.3389/fmolb.2025.1433912 (PMC11911207; doi:10.3389/fmolb.2025.1433912)

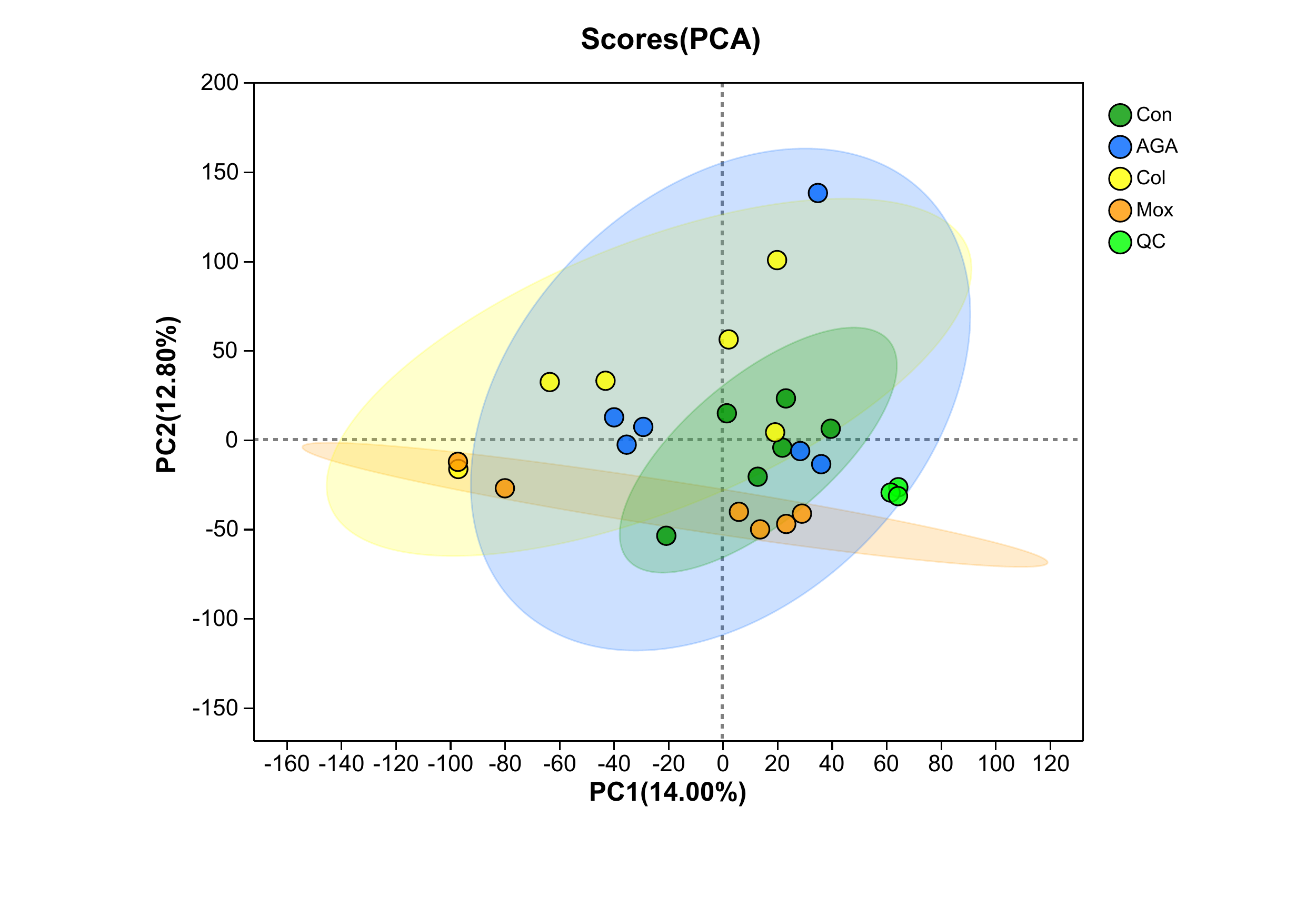

Supplement: Supplementary file 6 [file Image1.png]
